# A Comparative Study of the Functional Outcomes Between Distal Humerus Intra-articular Fractures Treated With Lateral Column Plate and Medial Screw Fixation Versus Bicolumnar Plate

**DOI:** 10.7759/cureus.96579

**Published:** 2025-11-11

**Authors:** Rohit Ajmeriya, Sachin Jain, Yogesh Singh Parihar

**Affiliations:** 1 Department of Orthopaedics and Traumatology, Gajra Raja Medical College, Gwalior, Gwalior, IND

**Keywords:** arc of motion, bicolumnar plate, intra-articular distal humerus fracture, lateral column plate, percutaneous medial screw

## Abstract

Background

Distal humerus fractures are complex injuries that pose significant challenges to orthopedic surgeons. Open reduction and internal fixation remain the mainstay of treatment. This study compared the clinical outcomes of intra-articular distal humerus fractures (excluding Arbeitsgemeinschaft für Osteosynthesefragen (AO) type 13-C3) treated with two fixation methods.

Methods

A total of 60 patients with intra-articular distal humerus fractures were included. Group I (n=30) underwent bicolumnar distal humerus plating, while Group II (n=30) underwent lateral column plating with intercondylar screw and percutaneous medial column screw fixation. Outcomes assessed included average arc of motion, union rate, and complications.

Results

In Group I, 18 patients (60%) achieved full arc of motion, 22 patients (73.3%) achieved fracture union, six patients (20%) developed infected non-union, and two patients (6.7%) had ulnar nerve palsy. In Group II, 28 patients (93.3%) regained full arc of motion, all achieved fracture union, and no major complications were observed.

Conclusion

Both fixation methods provided adequate stability and anatomic reconstruction; however, Group II demonstrated superior functional outcomes, higher union rates, and fewer complications compared to Group I.

## Introduction

Intra-articular distal humerus fractures remain challenging injuries for orthopedic surgeons. Open reduction and internal fixation (ORIF) continues to be the preferred surgical option for young and active patients who require good motor function. Anatomical restoration of the distal humerus facilitates early mobilization and aims to achieve a functional arc of motion.

A distal humerus fracture involves the articular portion of the elbow joint, including the lateral epicondyles, capitulum, and trochlea. These injuries compromise elbow movement and are most often caused by high-energy trauma such as road traffic accidents. In elderly patients with osteoporosis, even low-energy mechanisms, such as a simple fall, may result in fracture [1].

This is a relatively uncommon injury, accounting for approximately 2% of all fractures and one-third of humerus fractures, with an annual incidence in adults of about 570,000 cases. The injury demonstrates a bimodal age distribution, occurring most frequently in men aged 12-19 years and women over 80 years. In elderly patients, more than 60% of cases result from low-energy falls [2].

Currently, internal fixation with double plating (parallel or orthogonal) via a posterior approach - often with or without olecranon osteotomy - is considered to provide optimal outcomes due to precise reduction and maintenance of joint congruity [3]. However, double plating increases stiffness along the ulnar column, potentially limiting postoperative elbow motion [4].

While single lateral column plating alone is insufficient, combining it with an oblique medial column screw has been proposed to maintain stability. The complex anatomy of the medial column often makes optimal screw placement difficult, contributing to fixation failure [5].

ORIF using a posterolateral plate in combination with intercondylar and medial column screws (instead of a medial plate) has been described in the literature, with promising clinical outcomes. This prospective study aimed to compare the mechanical stability, functional outcomes (range of motion and early return to activities of daily living), radiographic results, complications, and operative time between bicolumnar plating and lateral column plating with medial and intercondylar screw fixation.

Patients were followed for six months, with serial radiographic assessment for fracture union, measurement of range of motion using the Mayo Elbow Performance Score (MEPS), monitoring of complications, and evaluation of changes from the preoperative period through follow-up.

Study objectives and hypothesis

The present study aimed to compare the clinical and radiological outcomes of intra-articular distal humerus fractures (Arbeitsgemeinschaft für Osteosynthesefragen (AO) type 13-C1 and 13-C2) treated with two fixation methods - bicolumnar plating and lateral column plating with medial and intercondylar screw fixation.

The primary objective of this study is to evaluate and compare the functional outcomes between the two fixation techniques using the MEPS and range of motion (ROM) at six months and secondary objectives are to compare operative duration and time to fracture union between the two groups, assess postoperative complications (infection, non-union, ulnar nerve palsy) and recovery of activities of daily living (ADLs).

The study tested the hypothesis that single lateral column plating with medial and intercondylar screw fixation provides better clinical and radiological outcomes than conventional bicolumnar plating in the management of intra-articular distal humerus fractures with null hypothesis (H_0_) - There is no significant difference in operative time, rate of fracture union, range of motion, or functional outcomes (MEPS) between patients treated with bicolumnar plating and those treated with lateral column plating with medial and intercondylar screws - and alternative hypothesis (H_1_) - Lateral column plating with medial and intercondylar screw fixation results in superior functional outcomes, shorter operative duration, earlier fracture union, and fewer complications compared to bicolumnar plating in intra-articular distal humerus fractures.

## Materials and methods

Study setting and design

This prospective randomized study was conducted in the Department of Orthopaedics and Trauma Centre, Jaya Arogya Group of Hospitals, Gwalior, Madhya Pradesh, India. The study commenced in May 2020.

Sample size and group allocation

A total of 60 patients with intra-articular distal humerus fractures were enrolled. Patients were randomly assigned into two groups using simple randomization: Group I (n=30): Treated with bicolumnar plating; Group II (n=30): Treated with single lateral column plating combined with intercondylar screw and percutaneous medial column screw fixation. Even-numbered patients were assigned to Group I, and odd-numbered patients to Group II. Patients were randomly assigned to two groups using a simple alternate allocation (even-odd sequence) method for feasibility. Although this provided balanced group sizes, it does not ensure true randomization. Therefore, in future studies, computer-generated randomization methods are recommended to minimize allocation bias.

Inclusion criteria

The inclusion criteria were as follows: patients aged between 18 and 60 years, having AO type 13-C1 or 13-C2 intra-articular distal humerus fractures, fractures requiring internal fixation, those medically fit for surgery, and those able to provide informed consent.

Exclusion criteria

The exclusion criteria were: refusal to provide consent, age <18 or >60 years , extra-articular fractures, pathological fractures, fractures with neurovascular compromise, grade III compound fractures, AO type 13-C3, or comminuted multifragmentary fractures.

Follow-up protocol

Patients were followed at 15 days, one month, three months, and six months postoperatively. Only cases with complete clinical records and radiographs were included in the final analysis. The mechanism of injury, fracture site, and fracture characteristics were recorded. AO classification was used preoperatively, and functional outcomes were assessed using the MEPS during follow-up.

Surgical procedure

Patient Positioning and Exposure

All surgeries were performed under aseptic precautions and prophylactic antibiotic coverage. Patients were positioned in the lateral decubitus position with the arm supported to allow 120° of elbow flexion. A posterior midline longitudinal incision (6-7 cm, extending 5 cm proximal and 1 cm distal to the elbow joint) was used, curving medially or laterally over the olecranon. The ulnar nerve was identified, released through the cubital tunnel, and retracted medially without tension.

Approach and Reduction Technique

Paratricipital exposure was used to access both columns. In cases requiring olecranon osteotomy, a chevron osteotomy was performed to enhance visualization. The triangle-of-stability principle was followed: the intra-articular fracture was reduced first (using K-wire joysticks, reduction forceps, or lag screws), converting it into an extra-articular fracture. The lateral column was usually fixed first. In Group I patients, bicolumnar plating (parallel or orthogonal configuration) with 3.5 mm locking compression plates was used. In Group II, fixation was performed using a lateral column locking compression plate combined with a percutaneous oblique medial column cancellous screw (4.0 mm). Further, an intercondylar lag screw was used selectively in cases with a visible intercondylar fracture line requiring compression across the articular surface. In fractures where anatomic reduction was achieved and maintained through the plate and medial screw construct, a separate intercondylar screw was not inserted.

The choice was made intraoperatively based on fracture configuration, stability after reduction, and surgeon discretion.

Biomechanics

**Figure 1:**
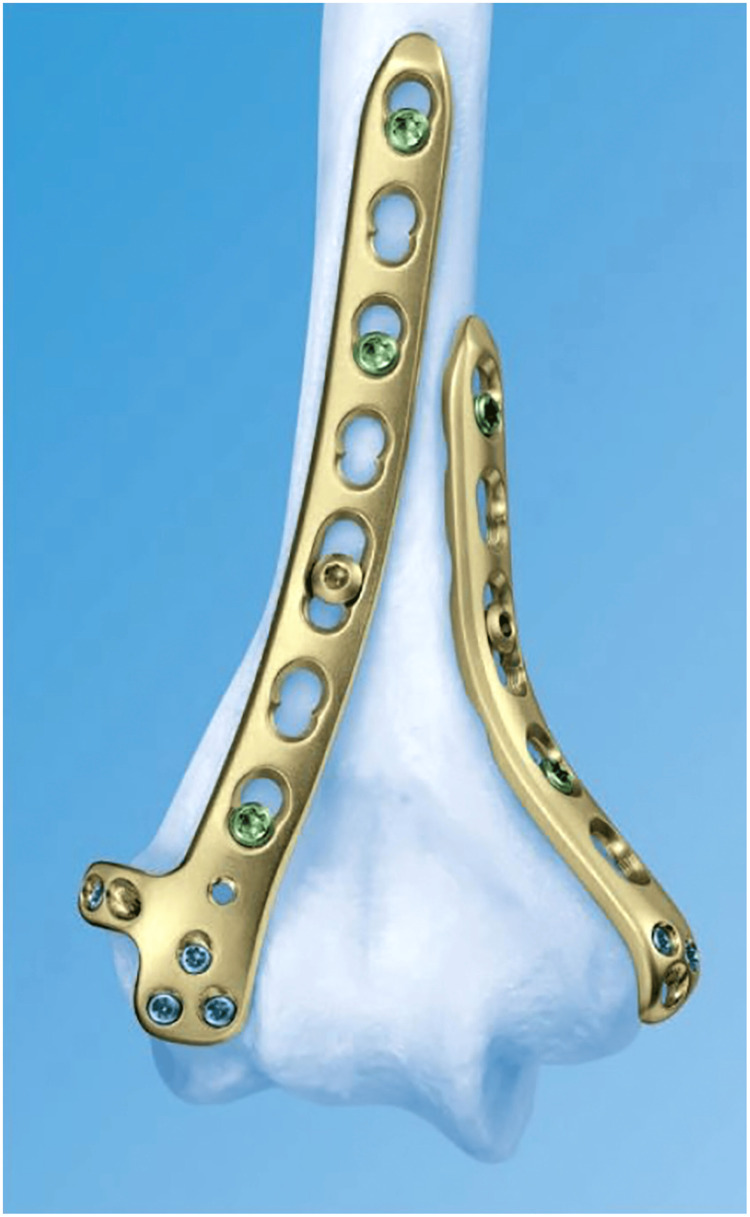
Bicolumnar Plate

The bicolumnar plate is shown in Figure 1.

Double plating provides superior stiffness and reduced displacement under axial compression, varus bending, and torsional forces. Orthogonal plating improves resistance to varus torsion, while parallel plating offers greater resistance to varus and bending forces. These configurations maintain joint congruity and are particularly effective in managing high-load stresses experienced by the elbow during functional activities.

**Figure 2:**
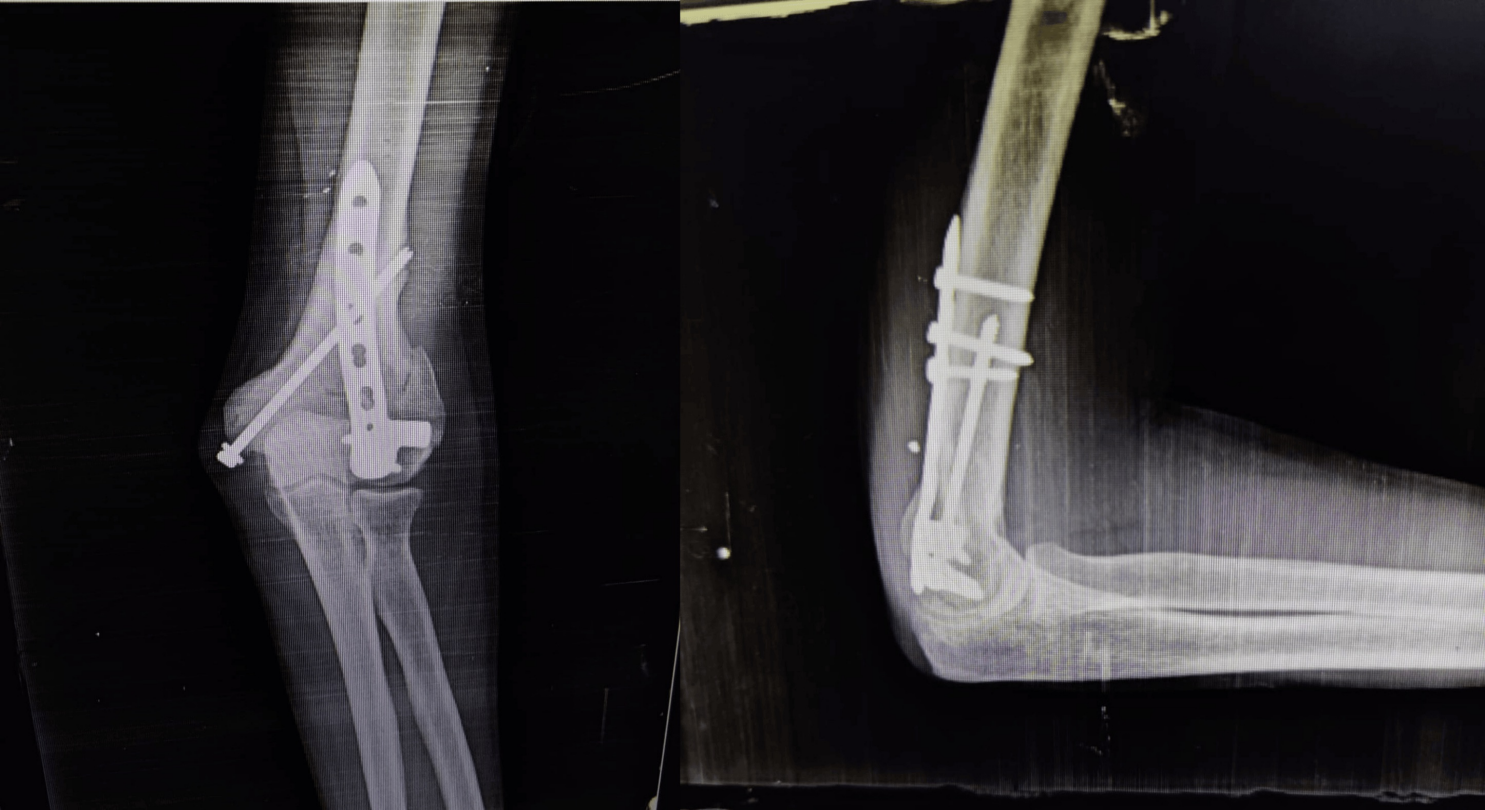
Lateral columnar plate with medial screw (LCPMS)

The lateral column plate with medial screw (LCPMS) is shown in Figure 2.

A single lateral column plate primarily resists varus loading but may be insufficient alone. Stability is enhanced by combining it with an oblique medial column screw, which acts intramedullary to support the medial column while allowing controlled micromotion. This approach reduces the risk of postoperative stiffness associated with medial plate fixation, while retaining the mechanical principle of bicolumnar stability through a lateral extramedullary plate and medial intramedullary screw construct.

The conventional bicolumnar plating technique provides rigid dual-column stability and high resistance to axial, torsional, and varus-valgus stresses, as demonstrated in prior biomechanical studies. However, excessive construct stiffness and soft-tissue dissection may contribute to postoperative stiffness and ulnar nerve morbidity. The lateral column plate combined with a percutaneous oblique medial column screw represents a hybrid extramedullary-intramedullary construct designed to achieve balanced dual-column support while allowing limited micromotion conducive to early mobilization. Biomechanical evaluations have shown that the addition of transcondylar or linking screws can enhance torsional and bending stability of distal humerus constructs without requiring a full medial plate. Furthermore, comparative finite-element and cadaveric studies have demonstrated that modified single-plate or linked constructs may achieve near-comparable stiffness to traditional bicolumnar plating in selected fracture types.

While these studies support the mechanical feasibility of the lateral plate-medial screw configuration, we acknowledge that dedicated biomechanical validation of the exact construct used in this study is warranted to quantify its stiffness, load-to-failure, and fatigue characteristics.

Postoperative rehabilitation

Active elbow flexion, extension, pronation, and supination exercises were initiated on postoperative day 2-3 within pain limits. Hand-pumping and forearm rotation exercises were encouraged early to reduce edema. Gravity-assisted active-assisted elbow exercises were performed hourly, and the arm was kept elevated between sessions for the first 48 hours.

Outcome measures

At each follow-up visit, the following parameters were recorded: Time to fracture union (radiological and clinical), arc of motion (elbow flexion-extension), complications, functional outcome using MEPS at 15 days, one month, three months, and six months, or until union.

Statistical analysis

Data collected from both groups were compiled and analyzed using IBM SPSS Statistics for Windows, Version 25.0 (IBM Corp., Armonk, NY, USA). Continuous variables such as age, operative duration, ROM, and MEPS were expressed as mean±standard deviation (SD).

Differences between the two groups for continuous variables were analyzed using the independent Student’s t-test, while categorical variables such as union rate, complication frequency, and activities of daily living (ADL) recovery were compared using the chi-square (χ²) test or Fisher’s exact test where applicable. A p-value of <0.05 was considered statistically significant for all comparisons. The data were further interpreted with 95% confidence intervals to support the strength of associations.

A post-hoc power calculation was conducted based on the difference in mean MEPS between the two groups using the observed standard deviations. With an alpha level of 0.05 and a two-tailed independent t-test, the achieved power (1-β) was 0.84, indicating an adequate probability of detecting the observed group difference as statistically significant. Therefore, the present sample size (n=60) was considered sufficient to support the main outcome comparison.

## Results

A total of 60 patients were analyzed, with 30 in Group I (bicolumnar plating) and 30 in Group II (lateral column plate with intercondylar and medial screw). Two patients in Group II were lost to follow-up and excluded from the final comparative analysis. The mean age was comparable between the groups (Group I: 38.04±11.97 years; Group II: 38.20±10.98 years; independent t-test, p=0.542), indicating no baseline age difference.

Operative duration

The mean operative duration was significantly shorter in Group II, with 60% of cases completed within 90-120 minutes compared to only 13.3% in Group I (Table 1).

**Table 1:** Comparison of the duration of surgery

Duration (minutes)	Group I: Bicolumnar Plating	Group II: Lateral Plate + Medial and Intercondylar Screw
90-120	4 (13.3%)	18 (60.0%)
120-150	26 (86.7%)	12 (40.0%)
Total	30 (100%)	30 (100%)

Range of motion

At six months, Group II achieved a significantly greater mean arc of motion (119±16°, range 90-130°) than Group I (100±10°, range 85-110°) (Table 2).

**Table 2:** A comparison of range of motion (ROM) between Group I and Group II Independent t-test, p<0.001.

ROM category	Group I	Group II	t-value	p-value
30-50°	8 (26.7%)	0 (0.0%)		
Up to 90°	17 (56.7%)	20 (66.7%)		
>100°	5 (16.6%)	8 (26.7%)	-5.01	<0.001*

Functional outcome: MEPS score

Group II had a higher mean MEPS score (93.5) compared to Group I (79.5) (t-test, p<0.001). Excellent-to-good outcomes were observed in 93.3% of Group II patients and 66.7% of Group I patients (Table 3).

**Table 3:** MEPS score distribution t-test, p<0.001.

Outcome	Group I: Bicolumnar Plating	Group II: Lateral Plate + Medial and Intercondylar Screw	p-value
Excellent	14 (46.7%)	24 (85.7%)	
Good	6 (20.0%)	4 (14.3%)	
Fair	6 (20.0%)	0 (0.0%)	
Poor	4 (13.3%)	0 (0.0%)	0.001*

Fracture union

Union occurred earlier in Group II, with 57.1% uniting within 14-16 weeks compared to none in Group I. Delayed union (>24 weeks) occurred only in Group I.

Complications

Complications were significantly higher in Group I, with 20% non-union, 20% infection at the osteotomy site, and 6.7% permanent ulnar nerve palsy. Group II had no permanent nerve injuries or infections (Table 4).

**Table 4:** Complications in post operative patient

Complication	Group I, n (%)	Group II, n (%)
Non-union	6 (20.0)	0 (0.0)
Infection+Osteotomy site	6 (20.0)	0 (0.0)
Ulnar nerve palsy (permanent)	2 (6.7)	0 (0.0)
Transient ulnar nerve palsy	10 (33.3)	6 (20.0)

Activities of daily living (ADL) recovery

Group II patients returned earlier to ADLs, with significant differences in combing hair, dressing, and eating at all follow-up intervals (Table 5).

**Table 5:** ADL recovery at 6 months

Activity	Group I: Yes, n (%)	Group II: Yes, n (%)
Combing hair	20 (66.7)	28 (93.3)
Personal hygiene	30 (100.0)	28 (93.3)
Dressing (shirt/shoes)	22 (73.3)	28 (93.3)
Eating	24 (80.0)	28 (93.3)

Postoperative pain

At six months, most patients in both groups reported no pain, with Group II showing a higher proportion of pain-free cases (80.0% vs. 53.3%). Mild pain was reported more in Group I, while moderate pain was absent in Group II ( Table 6).

**Table 6:** Distribution of patients according to post-operative pain at six months

Post-Operative Pain	Group I, n (%)	Group II, n (%)	Total, n (%)
Mild	10 (33.3)	6 (20.0)	16 (26.7)
Moderate	4 (13.3)	0 (0.0)	4 (6.7)
No pain	16 (53.3)	22 (80.0)	38 (66.7)

Statistical summary

Independent t-tests were used for continuous variables (age, ROM, MEPS). Chi-square tests were used for categorical variables (union rates, complications, ADL recovery). Statistical significance was set at p<0.05. Group II consistently demonstrated shorter operative time, faster union, higher MEPS, greater ROM, and fewer complications compared to Group I.

## Discussion

Distal humerus fractures, especially intra-articular types, are technically demanding injuries due to the complex anatomy of the elbow joint and the need for stable fixation to enable early mobilization [1,2]. In recent years, ORIF with double plating - either parallel or orthogonal - has been considered the gold standard for most complex fracture patterns [3]. However, concerns about postoperative stiffness, prolonged surgical time, and complications related to medial column plating have encouraged exploration of alternative fixation methods [4,5].

In our study, patients treated with lateral column plating combined with intercondylar and medial screw fixation (Group II) demonstrated significantly better functional outcomes compared to bicolumnar plating (Group I) at six months. Group II achieved a higher mean arc of motion (119° vs. 100°) and MEPS scores (93.5 vs. 79.5, p<0.001). This difference is clinically important, as Morrey et al. [6,7] demonstrated that at least 100° of flexion and 90° of forearm rotation are required for most daily living activities.

Comparison with the literature

Our findings are consistent with Mondal et al. [8] and Kumar et al. [9], who reported mean ages in the mid-thirties and a male predominance, largely attributable to high-energy mechanisms such as road traffic accidents [10]. Both studies confirmed that early mobilization after stable fixation leads to superior functional outcomes. In our cohort, Group II achieved earlier recovery in ADLs, with significantly more patients able to comb hair, dress independently, and eat comfortably within one month postoperatively.

Several studies have examined plating configurations for distal humerus fractures. Atalar et al. [11] and Sanchez-Sotelo et al. [12] reported good to excellent outcomes in over 80% of cases using parallel plating, with mean arcs of motion ranging from 97° to 120°. Athwal et al. [13] similarly documented functional recovery but emphasized that ulnar nerve morbidity and olecranon osteotomy complications remain concerns. Our study supports these observations, as Group I had a higher incidence of permanent ulnar nerve palsy (6.7%), transient neuropraxia (33.3%), and infection at the osteotomy site (20%).

Olecranon osteotomy, while providing optimal articular exposure, is associated with delayed union, wound dehiscence, and implant-related complications [14]. In our study, all Group I patients required osteotomy, whereas only 33.3% of Group II did, contributing to the significantly shorter operative times and lower infection rates in Group II. It should be acknowledged that the unequal use of olecranon osteotomy between groups represents a potential confounder. The osteotomy, while providing better exposure, is associated with higher rates of wound complications and delayed union. This difference may partially explain the higher complication rate and delayed recovery observed in Group I.

Biomechanically, O’Driscoll [15] noted that daily activities place repetitive varus stresses across the elbow, tensioning the lateral column. The LCPMS construct offers lateral extramedullary and medial intramedullary stability while allowing micromotion at the medial column, potentially reducing stiffness without sacrificing stability - a hypothesis supported by our clinical results.

From a biomechanical standpoint, bicolumnar plating provides dual-column stability by placing rigid fixation along both the medial and lateral cortices, which enhances torsional and bending resistance. However, this construct increases construct stiffness and may predispose to postoperative stiffness, ulnar nerve irritation, and hardware-related soft-tissue complications due to extensive dissection. In contrast, the lateral column plate with medial and intercondylar screw construct achieves stability through a combined extramedullary-intramedullary principle. The lateral plate resists varus and axial loads, while the oblique medial screw functions as an internal strut supporting the medial column and permitting controlled micromotion, which favors callus formation and early mobilization. This may explain the superior postoperative range of motion and earlier ADL recovery observed in our study. Nevertheless, this approach is not without potential limitations. Achieving optimal medial screw trajectory requires careful intraoperative fluoroscopic guidance, and suboptimal screw placement may compromise medial column support. Additionally, in highly comminuted or osteoporotic fractures, the lack of a full medial plate could reduce resistance to valgus stress, limiting its use in such fracture configurations. Thus, while our findings favor the lateral column construct in selected cases, careful patient selection and surgical expertise remain essential for achieving optimal outcomes.

Statistical interpretation

The superiority of Group II in our study was supported by statistically significant differences in operative time (p=0.008), union time (p<0.001), arc of motion (p<0.001), MEPS score (p<0.001), and complication rates (p=0.014). While not all ADL outcomes reached statistical significance, the consistent numerical advantage in Group II reinforces its clinical utility.

Although some differences in ADL outcomes between the two groups did not reach statistical significance, the consistent numerical advantage in Group II carries important clinical implications. Patients treated with lateral column plating and medial screw fixation were able to perform essential daily tasks - such as combing hair, eating, and dressing - earlier and with greater ease.

This early functional independence, even in the absence of a statistically significant p-value, represents a clinically meaningful improvement for patients, as it reflects a quicker return to self-care and social activity. In orthopedic outcome research, especially in upper limb trauma, such functional gains often translate into better patient satisfaction and rehabilitation compliance, underscoring the value of clinical as well as statistical interpretation.

Therefore, the findings suggest that lateral column plating with medial screw fixation provides not only biomechanical stability but also a tangible improvement in early postoperative quality of life, which may not be fully captured by statistical testing alone.

Validation of hypotheses

The observations of this study clearly refute the null hypothesis (H₀), which stated that there would be no significant difference in operative duration, fracture union, range of motion, or functional outcomes between the two fixation techniques. The findings consistently demonstrated statistically and clinically superior results for lateral column plating with medial and intercondylar screw fixation (Group II) in terms of operative time, union rate, MEPS score, and functional recovery.

Consequently, the alternative hypothesis (H₁) is supported by the results, confirming that the lateral plate-medial screw construct provides improved clinical and radiological outcomes compared to conventional bicolumnar plating for intra-articular distal humerus fractures.

Limitations

The primary limitation of this study is the simple even-odd allocation method used for group assignment. While this approach was practical, it does not ensure full allocation concealment and could introduce selection bias, potentially influencing group comparability despite baseline demographic similarities. Secondly, the relatively small sample size (n=60) may have limited the statistical power to detect subtle but clinically relevant differences, particularly in ADL outcomes. As a result, some trends that appeared numerically superior in Group II did not reach statistical significance. Thirdly, being a single-center study conducted at a tertiary care hospital, the patient population and surgical expertise may not fully represent broader clinical practice, thereby limiting the generalizability of the findings to other institutions or demographic groups. Fourth, the six-month follow-up period was relatively short for evaluating long-term functional recovery, late complications, or implant-related issues, which may have influenced interpretation of long-term outcomes. Additionally, postoperative rehabilitation compliance and minor variations in surgical technique could not be completely standardized, potentially contributing to variability in functional outcomes. An additional limitation is the unequal use of olecranon osteotomy between the two groups (100% in Group I vs. 33.3% in Group II), which may have acted as a confounding factor affecting operative duration, complication rates, and early postoperative rehabilitation.

Although the mechanical concept of the lateral plate with medial screw is supported by existing biomechanical literature, this study did not include experimental mechanical testing of this specific construct. Future cadaveric or finite-element biomechanical investigations are recommended to confirm its comparative strength and failure modes relative to standard bicolumnar plating.

Future multicentric studies with larger sample sizes, computer-generated randomization, longer follow-up, and standardized rehabilitation protocols are needed to validate these findings and enhance their external applicability.

Clinical implications

Given its lower complication profile, shorter operative time, and faster return to ADLs, lateral column plating with intercondylar and medial screw fixation may be considered a viable alternative to traditional bicolumnar plating in appropriately selected intra-articular distal humerus fractures. However, larger randomized controlled trials with long-term follow-up are required to validate these findings. Although certain differences in ADL recovery between the two groups did not achieve statistical significance, the consistent numerical advantage observed in Group II indicates meaningful clinical benefit. Patients treated with the lateral column plate and medial screw construct were able to perform self-care activities, such as combing hair and dressing, earlier than those treated with bicolumnar plating. Early return to independence, even without statistical significance, represents a tangible improvement in postoperative quality of life. Moreover, the routine use of olecranon osteotomy in all Group I patients likely contributed to longer operative duration, higher infection rates, and delayed rehabilitation, thus influencing postoperative outcomes.

## Conclusions

Based on the findings of this prospective comparative study, lateral column plating combined with medial and intercondylar screw fixation (Group II) demonstrated superior outcomes compared to traditional bicolumnar plating (Group I) for intra-articular distal humerus fractures (AO type 13-C1 and C2). Group II achieved shorter operative time, earlier fracture union, greater ROM, higher MEPS, and fewer postoperative complications, resulting in a faster return to ADLs. Both fixation methods provided stable fixation, but the lateral plate-medial screw construct offered distinct advantages in terms of reduced surgical morbidity, improved early functional recovery, and enhanced rehabilitation outcomes.

Future multicenter randomized studies with longer follow-up and dedicated biomechanical validation are recommended to further substantiate these findings and evaluate long-term implant performance.
